# *Panax ginseng* root, not leaf, can enhance thermogenic capacity and mitochondrial function in mice

**DOI:** 10.1080/13880209.2020.1756348

**Published:** 2020-05-04

**Authors:** Su-hui Wu, Han-bing Li, Gen-Lin Li, Yue-juan Qi, Juan Zhang, Bai-yan Wang

**Affiliations:** a He-Nan University of Chinese Medicine, Zheng-Zhou, China; b Basic Medical College, He-Nan University of Chinese Medicine, Zheng-Zhou, China

**Keywords:** Thermogenesis, RMR, BAT, mitochondrial respiration

## Abstract

**Context:**

*Panax ginseng* C. A. Meyer (Araliaceae) root and leaf have always been considered in the traditional theory as *hot* and *cold* properties, respectively.

**Objective:**

To clarify the *hot* and *cold* properties of ginseng root and leaf from a thermodynamic viewpoint.

**Materials and methods:**

Thirty ICR male mice were randomly assigned to control (water), ginseng root group (GRP) and ginseng leaf group (GLP) with a concentration of 0.075 g/mL; the volume was 0.1 mL/10 g (body mass) per day by intragastric administration for 20 days. Ultra-Performance Liquid Chromatography (UPLC) was used to determine quality control through seven ginsenosides contained in ginseng root and leaf. Rest metabolic rate (RMR) and energy expenditure were monitored every 9 days by TSE System. At the 20^th^ day, serum T3 or T4, liver or brown adipose tissue (BAT) mitochondrial respiration were investigated.

**Results:**

The quality control of GRP and GLP were within requirements of 2015 China Pharmacopoeia. The RMR (mLO_2_/h) in GLP (47.95 ± 4.20) was significantly lower than control (52.10 ± 4.79) and GRP (55.35 ± 4.48). Mitochondrial protein concentration and respiration were significantly increased in GRP (BAT, 79.12 ± 2 .08 mg/g, 239.89 ± 10.24 nmol O_2_/min/g tissue; Liver, 201.02 ± 10.89, 202.44 ± 3.24) and decreased in GLP (BAT, 53.42 ± 3.48, 153.49 ± 5.58; Liver, 138.69 ± 5.69, 104.50 ± 6.25) compared with control.

**Conclusions:**

The *hot* and *cold* properties of ginseng root and leaf are correlated with thermogenic capacity and mitochondrial function of BAT and liver, which deserve to further research.

## Introduction

The property theory of Chinese herbal medicine (CHM) is considered as the core of Chinese medical theory. However, the underlying mechanisms of the properties in CHMs remain unclear, which in turn presents a barrier for the modernization of Chinese herbal medicine. Usually, people rely on the *hot* or *cold* properties of herbal drugs as guidance for the diagnosis, classification and clinical application of CHMs (Wang et al. 2016). Scientists have suggested that the properties of CHM are linked with the energy metabolism, especially the thermogenesis. They argue that hot property CHM can make the body to produce more heat, which is why the patient is more comfortable in the cold place, while cold property CHM can make the body produce less heat, which is why the patient is more comfortable in the warm place. It was tested by monitor the animal’s behaviour through a tri-zone temperature control plate (40 °C, 25 °C, and 15 °C, respectively), they found the mice prefer to 15 °C plate if the hot property CHM was given, and vice versa (Zhou et al. 2009; Zhao et al. 2011). This evidence reveals the link between thermogenesis and the properties of CHM; however, the CHM workings and patterns that affect the CHM thermogenesis of the body remain unclear.

Mammals produce heat via the following processes (Lowell and Spiegelman 2000): 1) obligatory thermogenesis, which includes the basal metabolic rate (BMR) and diet-induced thermogenesis produced in the liver, gut and white adipose tissue; 2) exercise thermogenesis produced by the skeletal muscle during random physical exercise; 3) adaptive thermogenesis, also referred to facultative thermogenesis (Solmonson and Mills 2016), which is regulated by brain nerve and mainly includes shivering thermogenesis and the selected thermogenesis mediated by uncoupling proteins (UCPs) locus on the mitochondria of brown adipose tissue (BAT).

The thermoregulation capacity of endotherms is easy influenced by the environment temperature. Thermoneutral zones (TNZ) are regarded as the range of ambient air temperatures, over which the BMR or resting metabolic rate (RMR) is maintained constant at the basal level (i.e., BMR as the lowest level for an endotherm to stay conscious; Cannon and Nedergaard 2011; Gordon 2012). It has been shown that BMR/RMR, which is more commonly measured, could contribute approximately 50% of daily energy expenditure (DEE; McNab 2002). Values for BMR and RMR usually differ by less than 10% and sometimes are used interchangeably.

The variations of RMR in animals can be affected by many factors such as body size and illness. Numerous scientists have used BMR and/or RMR to monitor the energy expenditure for distinguishing clinical diseases such as diabetes or obesity. At the tissue level, except for the body size variation in BMR, the most associated organs with the relatively sizeable metabolism are heart, intestine, liver, and kidneys (Li et al. 2010). At the cellular level, changes in metabolic rate, independent of whether they are mediated via nervous system or by endocrine factors, are mostly derived from changes in mitochondria function (Rolfe and Brown 1997). In particular, the mass-specific metabolism variation can be reflected by the mitochondria density differences and the enzymatic activity of potential metabolic pathways.

Brown adipose tissue (BAT) is very important because of its capacity to enhance energy expenditure (EE) and regulate the energy balance, which is why it has received strong scientific interest over recent years (Virtanen et al. 2009). It is strongly innervated by sympathetic nervous system and can burn off excess energy by uncoupling the oxidative phosphorylation via uncoupling protein 1 (UCP1) on inner mitochondrial membrane, thus causing the ATP production to decrease and energy to dissipate and be released as heat (Cannon and Nedergaard 2004; Oelkrug et al. 2015). As has been noted, this is one part of the adaptive thermogenesis. Under the cold circumstances, BAT generates most heat to prevent hypothermia (Zhang and Wang 2006, 2007). So, if the body feels cold, BAT thermogenic capacity is stimulated to produce more heat. During this period, the BAT mitochondrial oxidative phosphorylation is enhanced and state 4 respiration may be higher than before when the ADP is used up in an isolated system due to an uncoupling effect. Liver is another organ which can affect the thermogenesis by accelerating the mitochondrial oxidative phosphorylation occur in state 3 (defined as ADP-stimulated respiration) and 4 respiration.

Thyroid hormones (TH), which are secreted by the thyroid gland, are known to increase metabolism and are the well-established principal endocrine regulators of both obligatory and facultative thermogenesis in human and animals (Goglia et al. 2002; Psarra et al. 2006; Cioffi et al. 2013; Solmonson and Mills 2016). It has been suggested that thyroxine (T4) supplementation can increase resting metabolism (Johannsen et al. 2012). Thyroid hormones increase BAT thermogenesis after T4 is converted to T3 in rodents (Broeders et al. 2016). Consequently, it has been thought that thyroid hormones are molecular determinants of thermogenesis (Silvestri et al. 2005). Because of this, there is a possible connection between thyroid hormones and BAT activity in cold circumstances, which potentially occur through mitochondria (Cioffi et al. 2013). Mitochondria, which have been and continue to be the target of most studies on the calorigenic effects of TH, provide nearly 90% of energy supply in cells (Cioffi et al. 2013).

**Figure 1.**
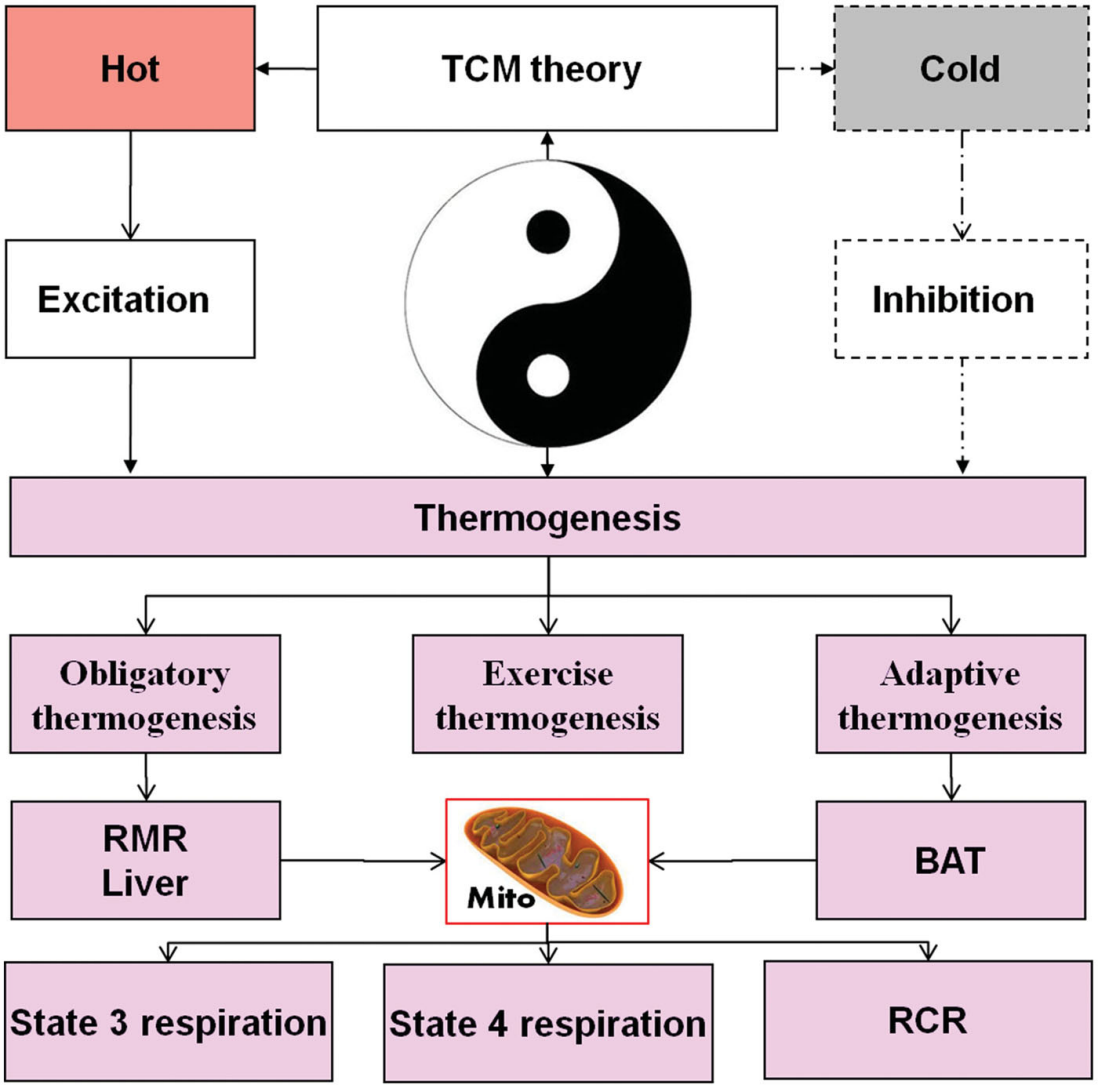
The thermodynamics hypothesis of TCM theory. The hot herb can make the body excitation so the thermogenesis increased and cold herb can make the body inhibition so the thermogenesis decreased.

*Panax ginseng* C. A. Meyer (Araliaceae) has been used for over 4000 years as a main constituent in many traditional Chinese medicine prescriptions for the prevention and/or treatment of diseases. In general, *P. ginseng* root has mainly been used due to its *hot* properties and important Tonifying-Qi function in treating ‘cold disease’, whose most obvious expression is that the body feels cold. Interestingly, the *P. ginseng* leaf, which is another part of ginseng, has been thought to have ‘cold’ properties and ability to let the body feels cold. According to Chinese Pharmacopoeia and some ancient books such as ‘New Compilation of Materia Medica’, which was first recorded by Yi-Luo Wu of the Qing dynasty, argue that the function of *P. ginseng* leaf has the function opposite to *P. ginseng* root. Until recently, scientists believed that the chemical composition of *P ginseng* leaf was similar to *P. ginseng* root and that both contained ginsenosides such as Re. Yet, it has been reported that ginseng root might be equally useful in improving cold tolerance in young and old animals (Wang and Lee 2000) and in recovery from acute hypothermia (Kumar et al. 1996). This might be because it increases consumption as a pharmacological agent and due to its influence on body temperature, particularly a ‘hot feeling’ (Cho et al. 2017). *P. ginseng* root crude extract has a stimulating effect on metabolism by significantly changing carbohydrate and lipid mobilization and utilization. Up to now, there is a lack of scientific evidence on the effects of ginseng leaf on the body and thermogenesis. Therefore, this study explores ‘hot’ and ‘cold’ properties in the ginseng root and leaf, respectively, by investigating the thermogenic effects of ginseng root on metabolic parameters such as RMR and energy expenditure, serum factors such as T3, T4, and mitochondrial respiration state. We assumed that hot herbs can induce the body excitation thus increasing the thermogenesis, while cold herb can lead to the body inhibition and decreased thermogenesis (as shown in Figure 1).

## Materials and methods

### Animals and traditional Chinese medicine

All the procedures that used mice in this experiment were approved by the institutional Animal Care and Use committee of Henan University of Chinese Medicine. Thirty CD-1 (ICR) male mice (SPF), weighing 30–35 g and 8 weeks old, used in this study were bought in the Vital River Laboratory Animal Technology Co., Ltd. Beijing, China. Animals were housed in groups of 3–4 in plastic cages (30 × 15 × 20 cm) with sawdust bedding in the animal house of Institute of Zoology. Food (standard pellets for mouse/rat, Beijing HFK Bioscience Co., Ltd. China.) and water were provided *ad libitum*. The mice were reared under 12 h light/dark cycle, with room temperature that was kept at 23 ± 1 °C. Before the beginning of the experiment, mice were allowed to acclimate for 1 week. Then all the animals were randomly assigned to control, ginseng root and ginseng leaf groups.

### Quality control determination of samples

The ginseng root and ginseng leaf were bought from Tongrentang Chinese medicine pharmacy in July, 2017, and were identified as the root and leaf of *P. ginseng* (Chinese Ginseng) by Professor Sui-qing Chen from Henan University of Chinese Medicine.

In order to ensure the quality of the samples and the controllability of traceability, the ginseng root and ginseng leaf were examined by Ultra Performance Liquid Chromatography (UPLC) to determine the content of ginsenoside Re, Rg1, Rb1, Rb2, Rf, Rc, and Rd. The experimental conditions were as follows: 1) Column: Waters Acquity BEH C_18_ column (100 mm × 2.1 mm, 1.7 µm); 2) chromatographic conditions were: flow rate 0.3 mL/min; detection wavelength (λ) = 203 nm; sample volume 5 µL; column temperature 30 °C. With acetonitrile (B) and 0.1% phosphoric acid solution (D) as mobile phase, gradient elution: 0–3 min, 19% B; 3–5 min, 20% B; 5–12 min, 21% B; 12–13 min, 29% B; 13–25 min, 31% B; 25–32 min, 35% B; 32–35 min, 19% B; 35–41 min, 19% B.3) Reference Standard: ginsenoside Re (lot number: 1205A021); Rg1 (lot number: 20160811); Rb1 (lot number: 2016530); Rb2 (lot number: 530A021); Rf (lot number: 817A021); Rc (lot number: 20151201); Rd (lot number: 20160816) were purchased from Beijing Solarbio Science Technology Co., Ltd. (http://www.solarbio.com/default.php).

The method of 2015 ‘China Pharmacopoeia’ fourth edition 9101 (analytical method validation) was used as a general reference for drug quality standard analysis to carry out the verification of guiding principles for determination of validation: 1) precision test: the RSD of the 7 main component peak areas were in the range of 0.80–0.91%; 2) repeated experiments: the chromatographic peak areas of 7 kinds of ginsenoside were in the range of 1.03–2.80%; 3) stability experiment: the RSD of 7 kinds of ginsenoside chromatographic peak area were in the range of 0.43–2.74%; 4) recovery: the recovery of 7 kinds of ginsenoside were in the range of 98.84–103.76%, the RSD value was between 1.23–2.72%, the results met the requirements.

### Animal experiment

We used 75.0 g of ginseng root and ginseng leaf, respectively, adding 600 mL water that was boiled for 30 min and the remaining water extract was drained out. Next, 450 mL water was added and left to boil for 30 min, after which the remaining water extract was drained out again. All water extract was put together, reaching the constant volume of 1000 mL. Finally, 0.075 g/mL concentrations of water extract of ginseng root and ginseng leaf were obtained. Importantly, the water extract of ginseng root and ginseng leaf were divided in vacuum bag, each one was kept at −20 °C for one day. Afterwards, the extracts were warmed to room temperature and shaken when used.

During experiment, the body mass was weighed at 08:00–10:00 every day at the same time. The intragastric administration was initiated with the volume of 0.1 mL/10 g (body mass). Cumulative body mass was calculated using the following formula:

Cumulative body mass=body mass at day n−body mass at day 0.

### Rest metabolic rate measurements

At day 0, day 9 and day 18 of the experiment, rest metabolic rates (quantified as oxygen consumption rate, VO_2_) and CO_2_ production (VCO_2_) were measured by an open-flow respirometry system (TSE Labmaster Calorimetry System, Germany) with an overall flow rate of 0.8 L/min and a sample flow rate of 0.39 L/min. RER were calculated as VCO_2_/VO_2_ (Arch et al. 2006). An incubator (Sanyo, MIR-554) was used to maintain the respiratory chambers (TSE, type I for mice, volume 2.7 L) at a constant temperature (30 ± 0.5 °C) within the TNZ of mouse (Pan et al. 2014). Real time ambient temperature in the incubator was recorded during metabolic measurements. All measurements were made between 08:00 and 20:00 h every day. Body weights were recorded before and after every measurement.

Each metabolic rate measurement lasted for 3 h. No fasting before measurement. Food and water were not provided during the measuring. RMR was calculated for each mouse as the average of two lowest constant oxygen consumption records (values of 5 min) at every temperature by using the data of last 2 h of metabolic rate measurement. Energy expenditure was recorded the same as RMR. RER was calculated using recordings of the average of two consecutive oxygen consumptions where the lowest was defined as RER_RMR_ (RER at the rest metabolic rate), and as RER_min_ (minimum RER during the entire metabolic rate measurement) was defined using recordings where the average of two consecutive RER values was the lowest.

### Serum thyroid hormone assays

In order to eliminate the effect of food, the animals were fasted 8 h and water *ad libitum* at the end of experiment. Then, at the 20 day of experiment, the mice were sacrificed by CO_2_ overdose. The trunk blood was collected 1 h later, and was centrifuged at 4000 rpm for 10 min, serum T3 and T4 were measured by ELISA kit (Nanjing Jiancheng Bioengineering Institute, China) according to the instructions. These kits were validated for mouse (reference). Intra- and inter-assay coefficients of variation were 11% and 9% for both.

### Mitochondria isolation and protein concentration determination

After collecting trunk blood, liver and inter-scapular-brown adipose tissue were dissected and weighted after fat or connective tissue were removed. Then the sample of liver was homogenized with ice-cold medium (5 mM Tris/HCL (pH 7.4), 250 mM sucrose and 2 mM EGTA), then centrifuged at 1000 *g* at 4 °C for 10 min, and the supernatant was centrifuged at 10,600 *g* at 4 °C for 10 min. This was repeated twice and the final deposit was resuspended with 0.1 mL isolation medium.

The BAT sample was weighed and then homogenized (1:15, w/v) with medium (10 mM TES, 250 mM sucrose, 1 mM EDTA, 64 10^−6^ mol/L BSA, pH 7.2). The homogenate was centrifuged at 12096 *g* at 4 °C for 10 min. Then, the supernatant was discarded, the precipitation with medium was resuspend (10 mM TES, 250 mM sucrose, 1 mM EGTA, 64 10^−6^ BSA, pH 7.2), centrifuged at 500 *g* at 4 °C for 10 min. The supernatant was then centrifuged at 8740 *g* at 4 °C for 10 min, and the last pellet was resuspended (1:1, w/v) with medium (20 mM TES, 100 mM KCl, 1 mM EGTA, pH7.2) and subsequently used for mitochondrial respiration. All medium was ice-cold.

Folin phenol method was used to determine total mitochondrial protein content and bovine serum albumin as standard (Zhao and Wang 2005; Li et al. 2010).

### Mitochondrial respiration

State 4 respiration was succinate dependent as the substrate in liver and BAT mitochondria. It was measured polaro-graphically using a Clark-type electrode (Hansatech Instruments, UK) at 30 °C, after which the ADP was added to determine state 3 respiration (Li et al. 2010; Pan et al. 2014).

### Statistical analyses

Data were analysed using SPSS 19.0 for windows software. Data were first examined for normality by Kolmogorov–Smirnov and homogeneity of variance by Levene tests. T3/T4 Ratio values were subjected to arcs in transfer for comparisons. To determine the group differences in BMR, energy expenditure, first we used one-way ANOVA analysis, and then the general linear modelling (GLM) with body weight as a covariate to decrease its potential effects on BMR. Pearson correlation was executed to examine possible associations between BMR and metabolism markers. Values are expressed as mean ± SE, *p* < 0.05 was considered to be statistically significant.

## Results

### Content determination of ginsenosides in ginseng roots and ginseng leaves

**Figure 2.**
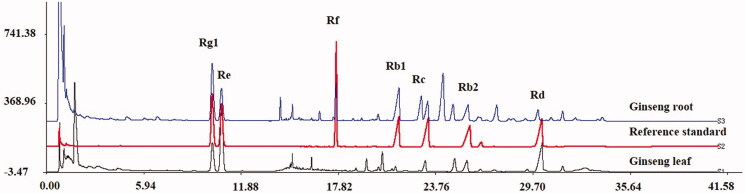
The UPLC of ginseng root and ginseng leaf.

The results of content determination (mg/g) of seven ginsenosides by Ultra Performance Liquid Chromatography (UPLC) were Rg1 (3.8482), Re (2.3623), Rf (1.0797), Rb1 (4.5249), Rc (1.9063), Rb2 (2.5465), Rd (0.7904) in ginseng root and Rg1 (13.0862), Re (32.5088), Rf (0), Rb1 (1.8325), Rc (5.4642), Rb2 (7.8677), Rd (20.7490) in ginseng leaves, respectively (Table 1; Figure 2). All the results were within requirements according to 2015 ‘China Pharmacopoeia’ fourth edition 9101 (analytical method validation). Consequently, the mice were treated with water extracts of ginseng root and ginseng leaf for further experimentation.

**Table 1.** The content determination of ginsenosides in ginseng roots and leaves (mg/g).

	Ginseng root		Ginseng leaf	
	Measured value	Mean	Measured value	Mean
Rg1	3.8056	3.8482	13.038	13.0862
	3.8605		13.1109	
	3.8785		13.1097	
Re	2.3469	2.3623	32.4923	32.5088
	2.3295		32.5132	
	2.4106		32.5207	
Rf	1.0757	1.0797	0	0
	1.0789		0	
	1.0843		0	
Rb1	4.6036	4.5249	1.8697	1.8325
	4.5099		1.8727	
	4.4613		1.7549	
Rc	1.9932	1.9063	5.4681	5.4642
	1.8347		5.4525	
	1.8909		5.472	
Rb2	2.5851	2.5465	7.8609	7.8677
	2.5867		7.8724	
	2.4678		7.8697	
Rd	0.7814	0.7904	20.7626	20.7490
	0.793		20.8245	
	0.7968		20.6599	

### Animal body mass change

The results of Repeated Measure ANOVA showed that the body mass of mice significantly increased with treatment (F_(3,81)_ = 33.898, *p* < 0.001), there was no significant difference among groups (F_(2,27)_ = 2.516, *p* = 0.100). However, the interaction between days and groups was statistically significant (F_(6,81)_ = 3.734, *p* = 0.002). The body mass at day 12 (*p* = 0.027) and 18 (*p* < 0.001) of the experiment significantly increased compared with the initial mice body mass.

**Figure 3.**
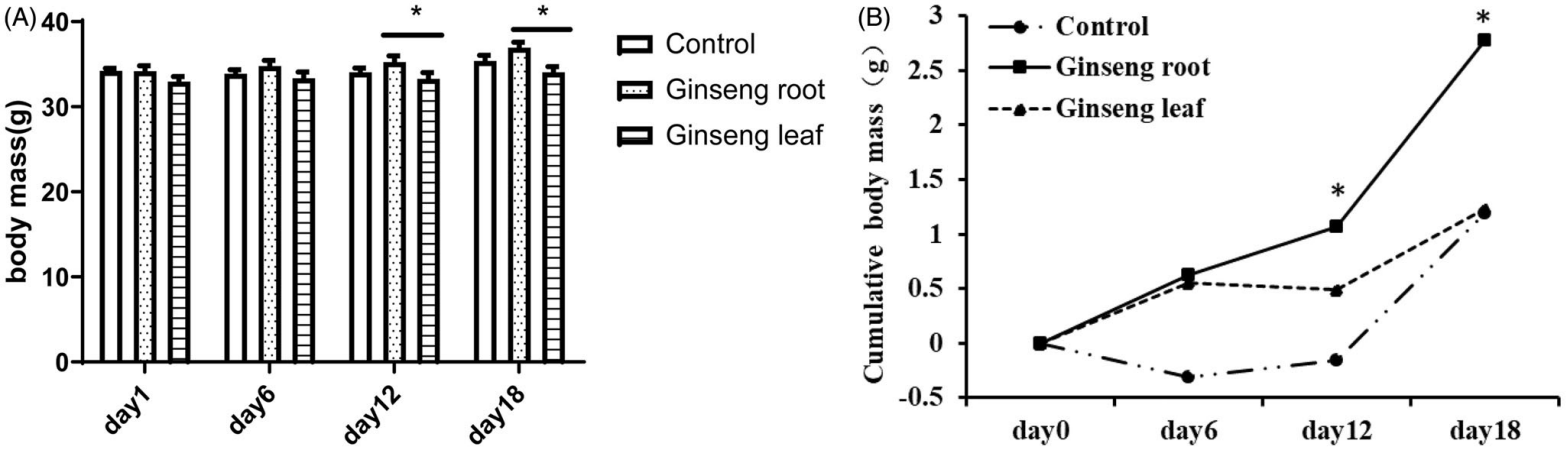
Changes of body mass (A) and cumulative body mass (B) of mouse during experiment. The bar represent mean ± SEM, the dot line represents mean of different group (*n* = 10). The dose is 7.5 g/Kg body mass of ginseng root and ginseng leaf group. At the day of 18, the body mass (one-way ANOVA, F_(2,27)_=4.205, *p* = 0.026) and cumulative body mass (one-way ANOVA, F_(2,27)_=5.200, *p* = 0.012) of water extract of ginseng root group and ginseng leaf group was affected significantly. There are significantly difference between ginseng root group and ginseng leaf group at the day of 12 (*p* = 0.048) and 18 (*p* = 0.007), The cumulative body mass of ginseng root group increased more, there are significantly difference between ginseng root group and ginseng leaf group at the day of 12 (*p* = 0.018) and 18 (*p* = 0.009). The symbol * represents *p* < 0.05 compared with ginseng root group.

The results of one-way ANOVA showed no significant difference among groups at the day 0 (F_(2,27)_ = 2.224, *p* = 0.128), day 6 (F_(2,27)_ = 1.374, *p* = 0.270), day 12 (F_(2,27)_ = 2.185, *p* = 0.132). At the day 18 of experiment, the body mass in mice were significantly different between groups (F_(2,27)_ = 4.205, *p* = 0.026). Moreover, the body mass of ginseng root group at day 12 (*p* = 0.048) and 18 (*p* = 0.007) of the experiment was significantly higher than in ginseng leaf group (multiple comparison; Figure 3(A)).

The cumulative body mass change reflect the body mass change rate. The data showed that the body mass in ginseng root and ginseng leaf group increased more during the experiment. The cumulative body mass at day 6 (F_(2,27)_ = 2.722, *p* = 0.084), and day 12 (F_(2,27)_ = 3.150, *p* = 0.059) of the experiment had no significant difference. However there was significant difference of cumulative body mass at day 18 (F_(2,27)_ = 5.200, *p* = 0.012) of experiment. As the experiment continued, the body mass change rate quickly increased, especially in ginseng root groups which were significantly higher than the control group at day 6 (*p* = 0.046), day 12 (*p* = 0.018), and day 18 (*p* = 0.009) (Figure 3(B)).

### Metabolic characteristics

**Figure 4.**
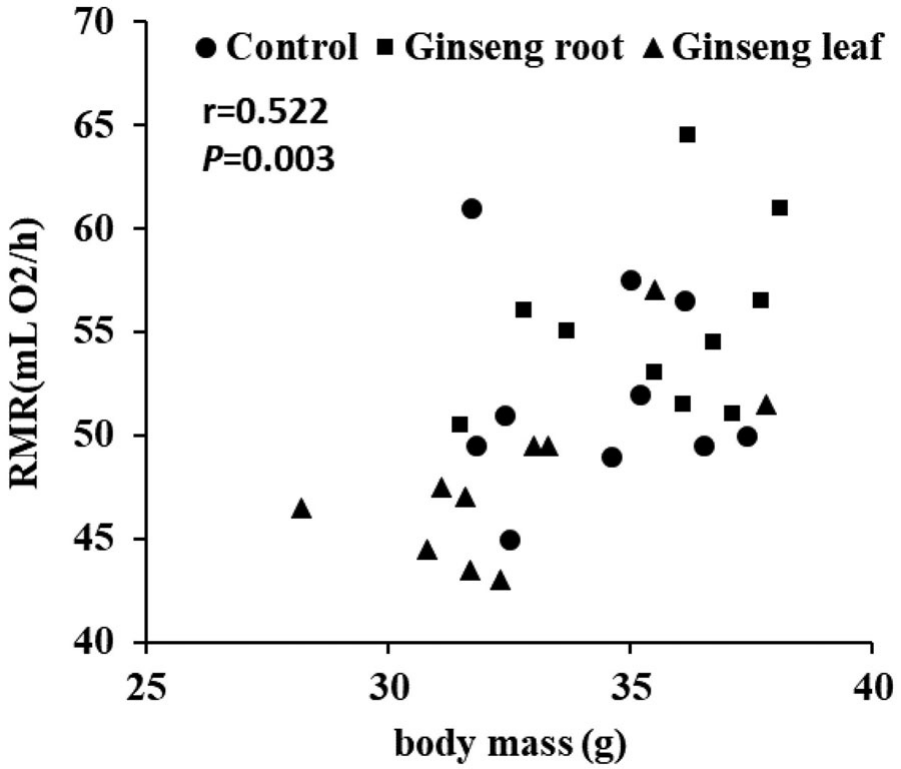
Relationships between RMR and body mass. Correlation analysis demonstrated that there was a strongly positive correlation between RMR (mLO_2_/h) and body mass (*r* = 0.522, *p* = 0.003), *p* < 0.05 was considered to be significantly correlated (Pearson correlation).

A significant difference in RMR (mLO_2_/h) was observed at day 9 (F_(2,27)_ = 3.485, *p* = 0.045) and 18 (F_(2,27)_ = 6.795, *p* = 0.004) of experiments among groups (Table 2). To test the association between RMR (mLO_2_/h) and body mass, we analysed this relationship with Pearson correlation. The results showed that the relationship between RMR (mLO_2_/h) and body mass had strong positive correlation (*r* = 0.522, *p* = 0.003; Figure 4).

**Table 2.** The effect of ginseng root and ginseng leaf on metabolic characteristics of mice (mean ± SE, *n* = 10).

	Control	Ginseng root	Ginseng leaf	*P* value
RMR (mLO_2_/h)				
Day 0	54.10 ± 3.18	54.75 ± 4.14	52.00 ± 3.13	ns
Day 9	49.75 ± 3.70^a^	53.75 ± 3.55^b^	49.65 ± 4.56^a^	0.045
Day 18	52.10 ± 4.79^a^	55.35 ± 4.48^a^	47.95 ± 4.20^b^	0.004
RMR (body mass-independent; mLO_2_/h/g)				
Day 0	1.57 ± 0.03	1.58 ± 0.03	1.52 ± 0.03	ns
Day 9	1.46 ± 0.03	1.54 ± 0.03	1.48 ± 0.03	ns
Day 18	1.48 ± 0.04^ab^	1.54 ± 0.04^a^	1.38 ± 0.04^b^	0.053
EE (body mass-independent; kcal/h/kg)				
Day 0	7.59 ± 0.14	7.62 ± 0.14	7.34 ± 0.15	ns
Day 9	7.01 ± 0.15	7.42 ± 0.16	7.11 ± 0.15	ns
Day 18	7.05 ± 0.18^ab^	7.41 ± 0.20^a^	6.66 ± 0.20^b^	0.056
RER_RMR_				
Day 0	0.83 ± 0.02	0.80 ± 0.02	0.82 ± 0.02	ns
Day 9	0.79 ± 0.01	0.80 ± 0.01	0.81 ± 0.01	ns
Day 18	0.79 ± 0.01	0.81 ± 0.01	0.81 ± 0.01	ns
RER_min_				
Day 0	0.75 ± 0.02	0.74 ± 0.01	0.74 ± 0.02	ns
Day 9	0.72 ± 0.01	0.73 ± 0.01	0.73 ± 0.01	ns
Day 18	0.72 ± 0.01^a^	0.75 ± 0.01^b^	0.74 ± 0.01^ab^	0.090

The data of body mass independent of RMR (mLO_2_/h/g), RMR (mLO_2_/h) and EE (kcal/h/kg) was presented in least square with body mass as a covariate (one-way ANCOVA). The different superscripts at the same row means that there are significantly differences with each other (*p* < 0.05).

To eliminate its potential effects on RMR, a general linear modelling (GLM) with body weight as a covariate was performed. The main significant differences in body mass of different groups was as follows: RMR (mLO_2_/h/g; One-way ANCOVA, body mass, F_(1,26)_ = 4.347, *p* = 0.047; Group, F_(2,26)_ = 3.288, *p* = 0.053), RMR (mLO_2_/h; One-way ANCOVA, body mass, F_(1,26)_ = 8.538, *p* = 0.007, Group, F_(2,26)_ = 3.114, *p* = 0.061) and EE (One-way ANCOVA, body mass, F_(1,26)_ = 4.854, *p* = 0.037; Group, F_(2,26)_ = 3.234, *p* = 0.056) at day 0, day 9 and day 18 of the experiment. The results of multiple comparisons showed that there were significant differences between ginseng root and ginseng leaf group in body mass-independent of RMR (mLO_2_/h/g), RMR (mLO_2_/h) and EE (kcal/h/kg) at day 18 of the experiment (Table 2).

### Serum thyroid hormones and thermogenesis

**Figure 5.**
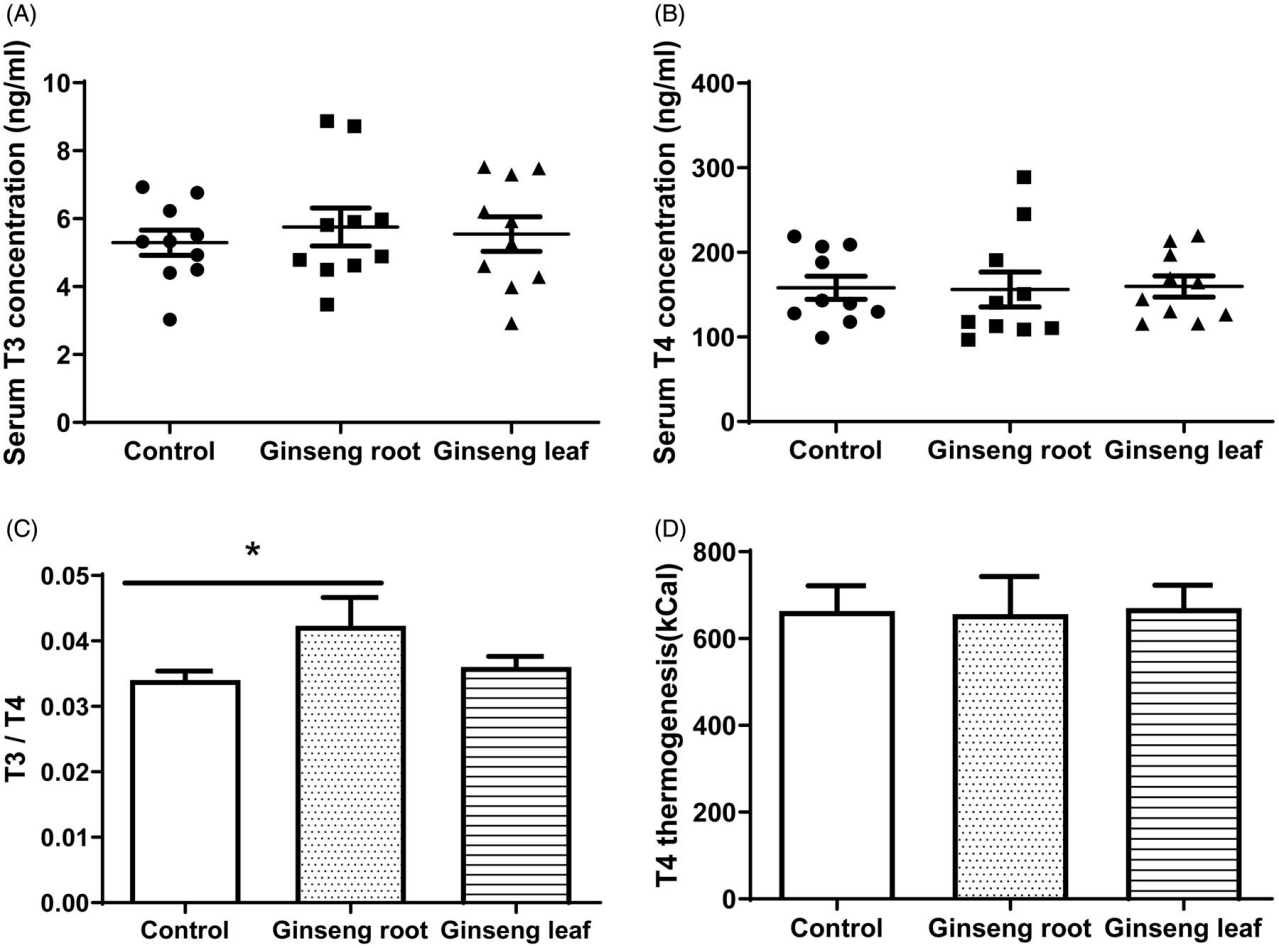
Serum T3 and T4 concentration, T3/T4 and T4 thermogenesis. The results showed that there was no significant difference in (A) serum T3 concentration (F_(2,27)_=0.224, *p* = 0.801), (B) T4 concentration (F_(2,27)_ =0.011, *p* = 0.989), T3/T4 ratio (F_(2,27)_ =2.403, *p* = 0.110) and T4 thermogenesis (F_(2,27)_ =0.011, *p* = 0.989).by one-way ANOVA. The symbol * represents *p* < 0.05 compared with control group (*p* < 0.05).

To explore the thermogenesis effect of ginseng root and ginseng leaf in mice, we detected the serum T3 and T4 concentration to reflect the linkage between thyroid hormones and thermogenesis. To avoid the potential effect of the kits’ and operation differences, we use the T3/T4 ratio to show thyroid hormones function. The results showed no differences in serum T3 concentrations (F_(2,27)_ = 0.224, *p* = 0.801, Figure 5(A)), T4 concentrations (F_(2,27)_ = 0.011, *p* = 0.989, Figure 5(B)) and T3/T4 ratio (F_(2,27)_ = 2.403, *p* = 0.110) by one-way ANOVA. However, the T3/T4 ratio in ginseng root group was significantly higher compared to control group (*p* = 0.045, Figure 5(C)).

In addition, it has been reported that mg T4 in the serum can generate approximately 4200 kcal of thermogenesis. Consequently, we calculated it to predict the thermogenesis of T4. The data showed no differences among groups (F_(2,27)_ = 0.011, *p* = 0.989, Figure 5(D)).

### Mitochondrial protein concentration and mitochondrial respiration in BAT and liver

There was no differences in wet weight of BAT among groups (F_(2,27)_ = 1.008, *p* = 0.378). Nevertheless, the BAT wet mass in ginseng root group and the ginseng leaf group were lower compared to control group. Interestingly, there was a significant difference of mitochondrial protein concentration (mg/g; F_(2,27)_ = 23.185, *p* < 0.001) and total mitochondrial protein (mg; F_(2,27)_ = 34.363, *p* < 0.001) between groups. Moreover, we compared State 4 Respiration of BAT in different groups. The data showed that either in protein-specific (nmol O_2_/min/mg Mt prot; F_(2,27)_ = 15.388, *p* < 0.001), mass-specific (nmol O_2_/min/g tissue; F_(2,27)_ = 29.144, *p* < 0.001) or total BAT activity (nmol O_2_/min in whole tissue; F_(2,27)_ = 7.095, *p* = 0.003) were significantly different among group. All of the above were significantly higher in ginseng root group compared to control group or ginseng leaf group (multiple compare).

Following the same trend, there was a significant difference of mitochondrial protein concentration (mg/g; F_(2,27)_ = 15.116, *p* < 0.001) among groups in the liver. Moreover, we also analysed liver’s State 3 and 4 Respiration in different groups. The data showed significant difference among groups either in protein-specific (nmol O_2_/min/mg Mt prot; F_(2,27)_ = 46.574, *p* < 0.001 for State 3 Respiration; F_(2,27)_ = 9.401, *p* = 0.001 for State 4 Respiration), mass-specific (nmol O_2_/min/g tissue; F_(2,27)_ = 90.174, *p* < 0.001 for State 3 Respiration; F_(2,27)_ = 11.295, *p* < 0.001 for State 4 Respiration) or respiration control rates (RCR; F_(2,27)_ = 8.932, *p* = 0.001).

All of the above were significantly higher in ginseng root group compared to control group or ginseng leaf group (multiple compare). Also, all of them in ginseng leaf group were lower compared to control group; however, the observed difference was not statistically significant except for the mass specific State 4 Respiration of BAT, mitochondrial protein concentration and mass specific State 3 Respiration of liver (Table 3).

**Table 3.** The effect of ginseng root and ginseng leaf to mitochondrial protein concentration (Mito. Prot. Conc.) and mitochondrial respiration (resp.) in liver and BAT of mice (mean ± SE, *n* = 10).

	Control	Ginseng root	Ginseng leaf	*P* value
BAT				
wet mass (g)	0.16 ± 0.01	0.14 ± 0.01	0.14 ± 0.01	0.378
mito.prot.conc. (mg/g)	53.33 ± 3.27^a^	79.12 ± 2.08^b^	53.42 ± 3.48^a^	<0.001
total mito.prot. (mg)	8.24 ± 0.17^a^	10.74 ± 0.31^b^	7.58 ± 0.34^a^	<0.001
State 4 Respiration				
nmol O_2_/min/mg Mt prot	7.91 ± 0.57^a^	12.23 ± 0.92^b^	7.29 ± 0.34^a^	<0.001
nmol O_2_/min/g tissue	187.90 ± 7.67^a^	239.89 ± 10.24^b^	153.49 ± 5.58^c^	<0.001
nmol O_2_/min in whole tissue	30.32 ± 2.64^a^	33.37 ± 2.30^a^	21.98 ± 1.56^b^	0.003
Liver				
mito.prot.conc. (mg/g)	168.28 ± 6.47^a^	201.02 ± 10.89^b^	138.69 ± 5.69^c^	<0.001
State 3 Respiration				
nmol O_2_/min/mg Mt prot	11.32 ± 0.33^a^	12.34 ± 0.44^a^	7.06 ± 0.46^b^	<0.001
nmol O_2_/min/g tissue	141.37 ± 5.64^a^	202.44 ± 3.24^b^	104.50 ± 6.25^c^	<0.001
State 4 Respiration				
nmol O_2_/min/mg Mt prot	6.98 ± 0.34^a^	5.28 ± 0.46^b^	4.71 ± 0.35^b^	<0.001
nmol O_2_/min/g tissue	87.15 ± 3.01^a^	86.47 ± 1.89^a^	71.70 ± 2.76^b^	<0.001
RCR	1.65 ± 0.09^a^	2.44 ± 0.22^b^	1.56 ± 0.14^a^	0.001

The different superscripts at the same row means that there are significantly different with each other (One-way ANOVA, *p* < 0.05).

### RMR and BAT or liver mitochondria protein concentration

**Figure 6.**
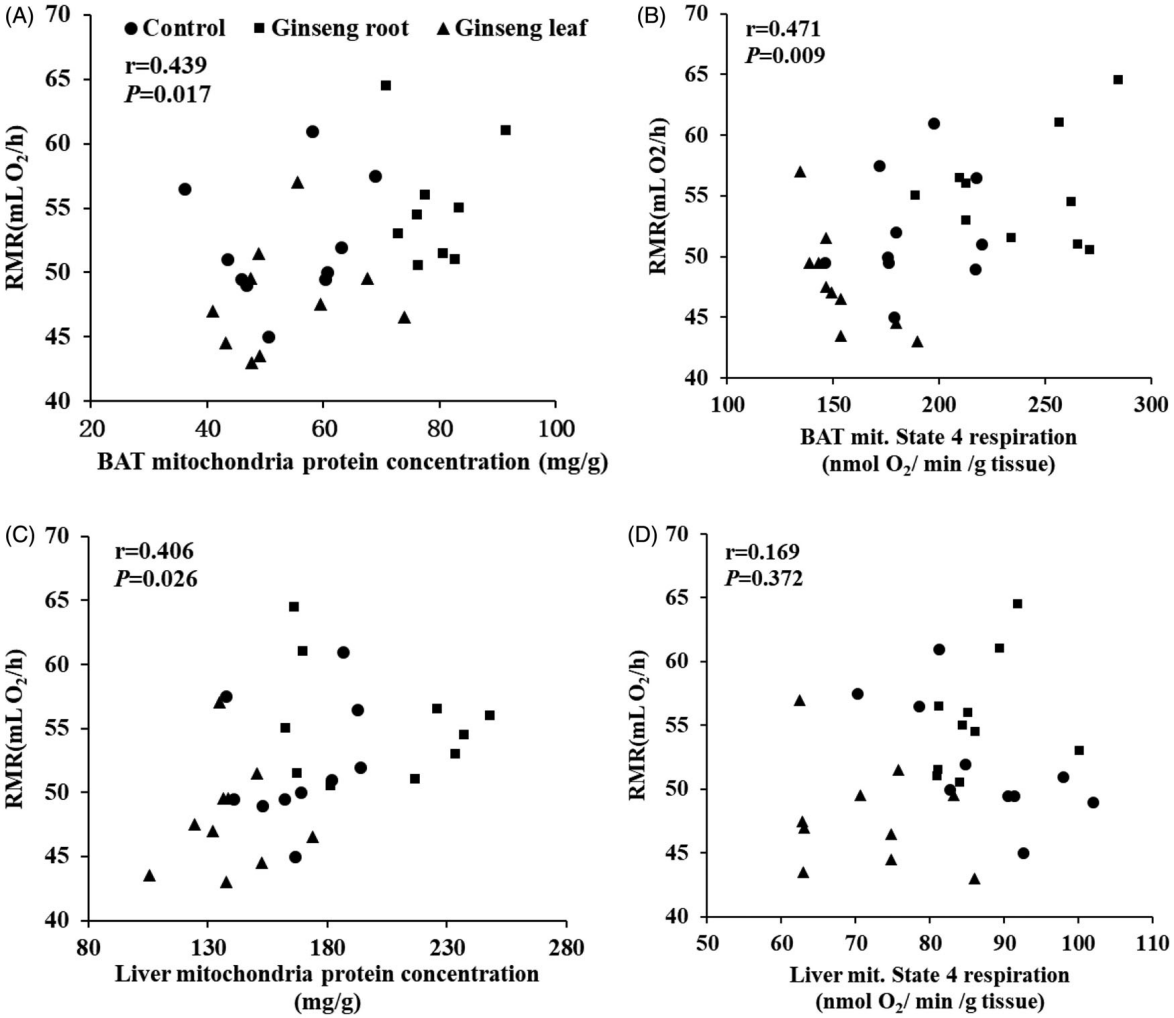
Relationships between rest metabolic rate and cellular and biochemical metabolic makers of BAT, liver and T4 themogenesis in mice. Correlation analysis showed that there were significant positive relationships between RMR and (A) BAT mitochondria protein concentration (*r* = 0.439, *p* = 0.017), (B) BAT mitochondria state 4 respiration (nmol O_2_/min/g tissue; *r* = 0.397, *p* = 0.040), (C) liver mitochondria protein concentration (*r* = 0.406, *p* = 0.026), (D) liver mitochondria state 4 respiration (nmol O_2_/min/g tissue; *r* = 0.169, *p* = 0.372). *p* < 0.05 was considered to be significantly correlated (Pearson correlation).

To explore the relationship between RMR and tissue function, we plotted the RMR (mL O_2_/h) against the BAT or liver mitochondria protein concentration, and mitochondria state 4 respiration. The results showed a positive significant correlation between RMR and BAT mitochondria protein concentration (*r* = 0.439, *p* = 0.017, Figure 6(A)), BAT mitochondria state 4 respiration (nmol O_2_/min/g tissue; *r* = 0.397, *p* = 0.040, Figure 6(B)), liver mitochondria protein concentration (*r* = 0.406, *p* = 0.026, Figure 6(C)), but no significant difference with liver mitochondria state 4 respiration (nmol O_2_/min/g tissue; *r* = 0.169, *p* = 0.372, Figure 6(D)).

### RMR and thyroid hormones

**Figure 7.**
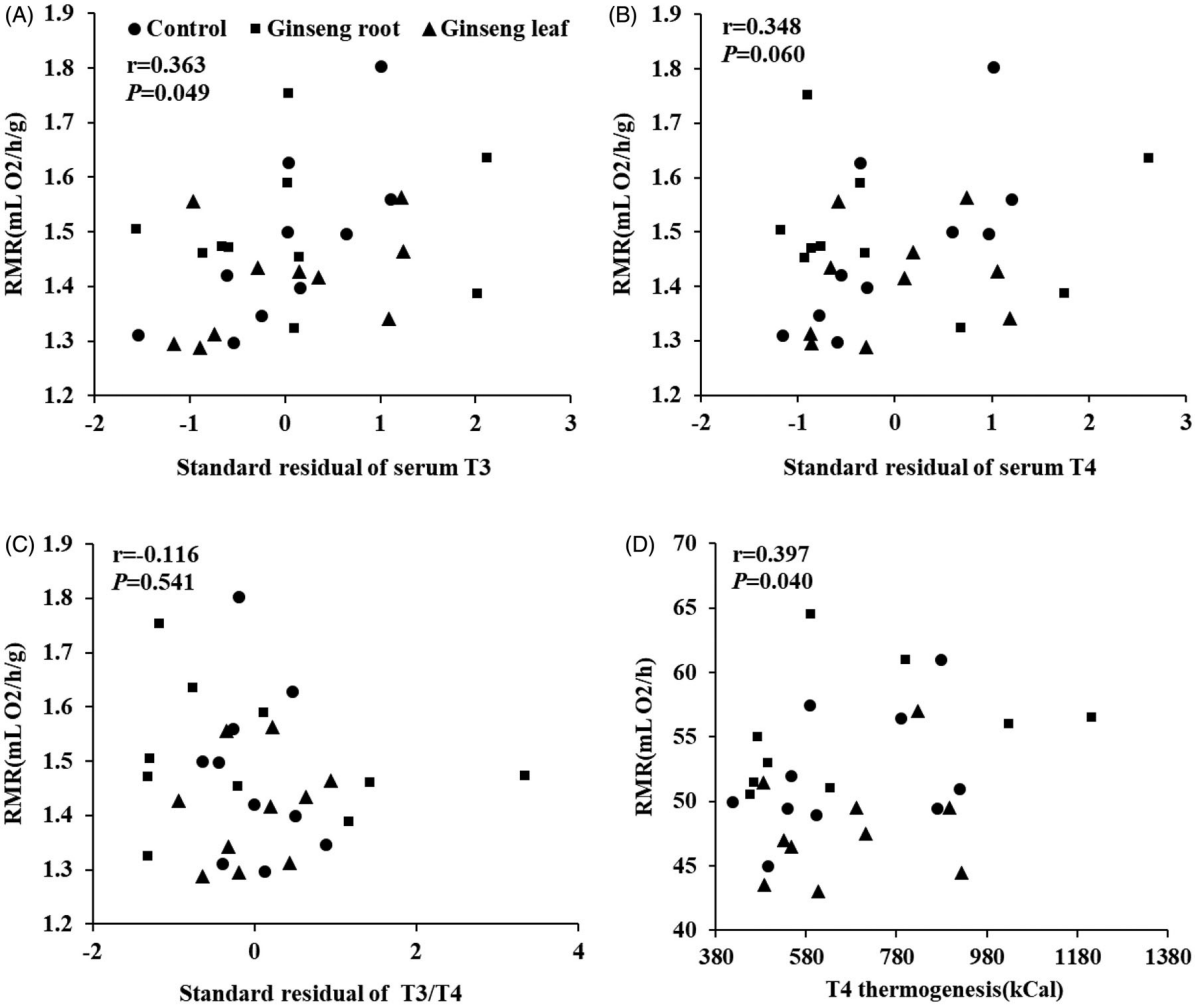
Relationships between rest metabolic rate and thyroid hormones. Correlation analysis showed that there were significant positive relationships between RMR and (A) standard residual of serum T3 (*r* = 0.363, *p* = 0.049), (B) standard residual of serum T4 (*r* = 0.348, *p* = 0.060), (C) standard residual of T3/T4 ratio (*r* = −0.116, *p* = 0.541), (D) T4 thermogenesis (*r* = 0.397, *p* = 0.040). *p* < 0.05 was considered to be significantly correlated (Pearson correlation).

In order to examine the relationship between RMR and thyroid hormones, we made a comparison among three groups. The results showed a significant positive correlation between RMR and standard residual of serum T3 (*r* = 0.363, *p* = 0.049, Figure 7(A)), and no significant relationship between RMR and standard residual of serum T4 (*r* = 0.348, *p* = 0.060, Figure 7(B)), and standard residual of T3/T4 ratio (r = −0.116, *p* = 0.541, Figure 7(C)). However, the relationship between RMR and T4 thermogenesis was significantly positive (*r* = 0.397, *p* = 0.040, Figure 7(D)).

### T3/T4 and BAT or liver mitochondria respiration

**Figure 8.**
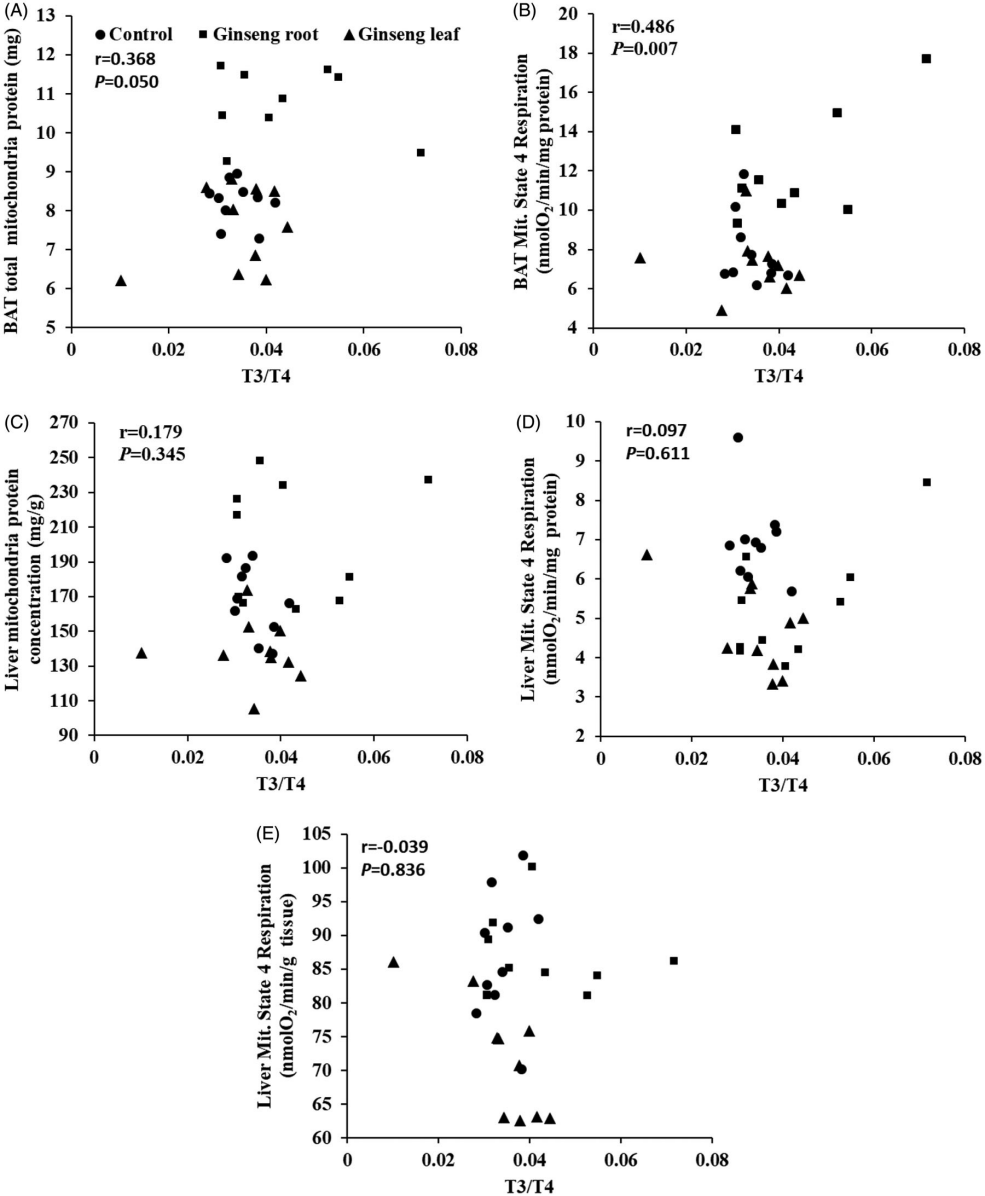
Correlations between the serum T3/T4 and (A) BAT total mitochondria protein (*r* = 0.368, *p* = 0.050), (B) BAT mitochondria state 4 respiration (*r* = 0.486, *p* = 0.007), (C) liver mitochondria protein concentration (*r* = 0.179, *p* = 0.345), (D, E) liver mitochondria state 4 respiration (nmolO_2_/min/mg mitochondria Protein, *r* = 0.097, *p* = 0.611; nmolO_2_/min/g tissue, *r* = −0.039, *p* = 0.836). *p* < 0.05 was considered to be significantly correlated (Pearson correlation).

To further study the possible causal relationship between the physiological and biochemical parameters, we plotted the metabolism markers against T3/T4. The results indicated a significant positive correlation between T3/T4 and BAT total mitochondria protein (*r* = 0.368, *p* = 0.050 Figure 8(A)), BAT mitochondria state 4 respiration (*r* = 0.486, *p* = 0.007, Figure 8(B)). There was no significant correlation between T3/T4 and liver mitochondria protein concentration (*r* = 0.179, *p* = 0.345 Figure 8(C)), liver mitochondria state 4 respiration (nmolO_2_/min/mg mitochondria Protein, *r* = 0.097, *p* = 0.611, Figure 8(D); nmolO_2_/min/g tissue, r = −0.039, *p* = 0.836, Figure 8(E)).

## Discussion

The *cold* or *hot* properties of herbal drugs are very important in Traditional Chinese Medicine (TCM) that has a long-standing tradition of nearly 4000 years. Numerous scientists have explored these properties from the aspect of endocrinological, immunological, neurological (Li et al. 1999), and thermodynamic aspects (Zhao et al. 2011). Since the concept of thermodynamics was proposed, it has become an important research topic among scientists. Previous thermodynamics studies focussing on the thermogenesis of animals have observed the thermotropism behaviour in Kunming mice with *cold* or *hot* syndrome under different circumstances. Their results suggested that the traditional ‘cold’ drug Coptidis could acutely decrease the energy metabolism and regulate animals’ response in preferring a warm environmental temperature. Meanwhile, the *cold* or *hot* drugs could change oxygen consumption, liver SOD activity, ATPase activity in mice (Zhao et al. 2011) and serum creatine kinase in rat, which suggest that these observations were related to the impact of herb medicine to energy metabolism (Zhang et al. 2009). In this paper, we investigated certain thermogenic parameters at tissue and cellular level to examine the essence of *cold* or *hot* properties.

A significant difference was found in RMR among groups; when giving the ginseng root to animals, the RMR increased, when giving the ginseng leaf to animals, the RMR decreased. Moreover, the RMR in ginseng root group was significantly higher compared to ginseng leaf group. When we tried to use GLM to account for the effect of body weight on RMR due to strongly significant positive correlation between RMR and body mass (*r* = 0.522, *p* = 0.003), the results of body mass-independent RMR (mL O_2_/h/g) and RMR (mL O_2_/h) were significantly higher in ginseng root group than in ginseng leaf group. In particular, we found that the energy expenditure measured by TSE increased in ginseng root group and decreased in ginseng leaf group compared with control group; the observed difference between ginseng root and ginseng leaf group was statistically significant. This evidence provides direct support for the hot property of ginseng root and cold property of ginseng leaf. During the experiment, the RER_RMR_ did not differ among groups, while RER_min_ was increased in ginseng root group compared to control group. This suggested that the ginseng root might induce the metabolic intensity of fatty tissue. We also detected the content determination (mg/g) of seven ginsenosides by UPLC method finding a very significant difference between the ginseng roots and leaves. It is possible that ginsenosides contribute to the above differences. These results are similar to those observed in North American ginseng root with reference to effectiveness of a particular active component that can significantly enhance the thermogenesis and cold resistance in rats (Wang and Lee 2000). It is also similar with the effect of red ginseng root that can increase VO_2_, VCO_2_, heat production and energy expenditure. The ginseng leaf group showed the opposite trend in hypothyroidism rats (Xiao et al. 2017). However, animal studies have shown that *Panax ginseng* (Korean ginseng) and *Panax quinquefolius* L. (American ginseng) have no effect on body temperature, VO_2_, VCO_2_, activity, food intake and energy expenditure after acute or chronic treatment with ginseng under normal conditions in mice (Park et al. 2014; Hong et al. 2015). This might be explained by geoherbalism which is important feature of Traditional Chinese Medicine.

Furthermore, we found that although the BAT wet mass did not differ among groups, the BAT mitochondrial protein concentrations (mg/g) and RMR (mL O_2_/h) were positively correlated (*r* = 0.439, *p* = 0.017). The similar positive correlation was also found between liver mitochondrial protein concentration (mg/g) and RMR (mL O_2_/h; *r* = 0.406, *p* = 0.026). We also found that liver contributed to some 20% of BMR, while metabolic intensity differences were linked with differences in oxidative capacities, mitochondrial densities and mitochondrial proton leaks (Rolfe and Brown 1997). The relationship between metabolic rate and mitochondrial respiration and leak was strongly correlated (Li et al. 2010). In this paper, we found statistically significant differences in mitochondrial protein concentration and total mitochondrial protein, state 4 respiration of BAT and mitochondrial protein concentration, state 3 and 4 respiration and RCR in liver. Moreover, we also analysed the correlations between RMR and these parameters; the results indicated that metabolic intensity of tissues might increase following the ginseng root treatment compared to the ginseng leaf treatment. The *hot/cold* property of ginseng root and leaf may greatly contribute to the differences in RMR and these biochemical metabolic makers of BAT and liver.

At the hormonal level, the T3 and T4 concentrations in serum did not differ among groups; however, the T3/T4 ratio in the ginseng root group was higher than in control groups. More importantly, there was a significant positive correlation between T4 thermogenesis and RMR (mL O_2_/h; *r* = 0.397, *p* = 0.040), which was consistent with the previous studies reporting that thyroid hormones are an important determinant of BMR (Mullur et al. 2014). Endogenous variation of T4 concentrations seems to be correlated with metabolic rate in animals (Li et al. 2010). It has been reported that T4 supplementation stimulate RMR in Brand’s voles, which is a small rodent inhabiting Inner Mongolia of China (Liu et al. 1997). We also found the positive correlation between standard residual T3, T4 and RMR (mL O_2_/h/g), suggesting that thyroid is an important hormone for the RMR thermogenesis. Moreover, the thyroid might contribute to the BAT adaptive thermogenesis, since the relationship between T3/T4 and BAT total mitochondrial protein (*r* = 0.368, *p* = 0.050), BAT mitochondrial state 4 respiration (*r* = 0.486, *p* = 0.007) was positively correlated. However, we did not find the significant relationship between T3/T4 and RMR (*r* = 0.088, *p* = 0.646), liver mitochondrial protein (*r* = 0.179, *p* = 0.345) and liver mitochondrial state 4 respiration (*r* = 0.039, *p* = 0.836), which suggests that BAT may have an important role in the determination of *hot/cold* property for ginseng root and leaf. As is well known, the thermogenic capacity of BAT mostly depends on UCP1, which can make the proton gradient across the inner mitochondrial membrane dissipate (Endo and Kobayashi 2008) and protect the mitochondria against the enhanced risk of radical production and oxidative damage during oxidative phosphorylation. Previous studies have suggested that thyroid hormones adapt for mitochondrial uncoupling to stimulate the metabolic rate and decrease the metabolic efficiency (Lanni et al. 2003). This suggests that the Tonifying-Qi function of ginseng root at a chronically high doses can cause inflammation such as the ginseng-abuse syndrome (Xu and Dou 2016). Consequently, future studies should explore the differences in uncoupling proteins (UCPs), proton conductance and the mitochondrial membrane composition when using ginseng root and leaf to establish the mechanisms of the *hot/cold* property.

## Conclusions

The above reported results showed that the *cold and hot* properties of herb medicines are correlated with body energy conditions and regulation, which confirmed our initial hypothesis. The *cold* herbs down-regulate, while *hot* herbs up-regulate thermogenesis. In particular, this is the first report that used BAT thermogenesis and mitochondrial respiration to study the traditional theory of *hot* and *cold* herbal drugs. Thanks to the physiological and biochemical analysis, this method may provide a unique insight in the research of herbal drugs, and can also be applied for research and study of other herbs.
